# Deafness and loss of cochlear hair cells in the absence of thyroid hormone transporters Slc16a2 (Mct8) and Slc16a10 (Mct10)

**DOI:** 10.1038/s41598-018-22553-w

**Published:** 2018-03-13

**Authors:** David S. Sharlin, Lily Ng, François Verrey, Theo J. Visser, Ye Liu, Rafal T. Olszewski, Michael Hoa, Heike Heuer, Douglas Forrest

**Affiliations:** 1 Department of Biological Sciences, Minnesota State University Mankato, Mankato, Minnesota 56001 USA; 2 Laboratory of Endocrinology and Receptor Biology, NIDDK, National Institutes of Health, Bethesda, Maryland 20892 USA; 3 Center for Integrative Human Physiology (ZIHP) and NCCR Kidney. CH, Institute of Physiology, University of Zürich, Zürich, 8057 Switzerland; 4 Department of Internal Medicine and Rotterdam Thyroid Center, Erasmus University Medical Center, Rotterdam, The Netherlands; 5 National Institute on Deafness and other Communication Disorders, National Institutes of Health, Bethesda, Maryland 20892 USA; 6 Department of Endocrinology, Diabetes and Metabolism, University Hospital Essen, University of Duisburg-Essen, 45147 Essen, Germany

## Abstract

Transmembrane proteins that mediate the cellular uptake or efflux of thyroid hormone potentially provide a key level of control over neurodevelopment. In humans, defects in one such protein, solute carrier SLC16A2 (MCT8) are associated with psychomotor retardation. Other proteins that transport the active form of thyroid hormone triiodothyronine (T3) or its precursor thyroxine (T4) have been identified *in vitro* but the wider significance of such transporters *in vivo* is unclear. The development of the auditory system requires thyroid hormone and the cochlea is a primary target tissue. We have proposed that the compartmental anatomy of the cochlea would necessitate transport mechanisms to convey blood-borne hormone to target tissues. We report hearing loss in mice with mutations in *Slc16a2* and a related gene *Slc16a10* (*Mct10*, *Tat1*). Deficiency of both transporters results in retarded development of the sensory epithelium similar to impairment caused by hypothyroidism, compounded with a progressive degeneration of cochlear hair cells and loss of endocochlear potential. Administration of T3 largely restores the development of the sensory epithelium and limited auditory function, indicating the T3-sensitivity of defects in the sensory epithelium. The results indicate a necessity for thyroid hormone transporters in cochlear development and function.

## Introduction

Neurodevelopment is known to require adequate thyroid hormone in the circulation but for many years, little attention was given to questions of access of the hormone to target tissues^1,2^. The necessity for proteins that transport hormone across the plasma membrane of cells was indicated by finding mutations in solute carrier SLC16A2 (monocarboxylate transporter 8, MCT8) in Allan-Herndon-Dudley syndrome, an X-linked disorder of psychomotor and speech retardation^3,4^. SLC16A2 has 12 transmembrane domains and transports the active form of thyroid hormone, T3, and its precursor T4^5^. *In vitro* studies have identified other proteins that transport T3 or T4 among other substrates, suggesting that the current picture of thyroid hormone transport is far from complete^6^. Moreover, it has been suggested that Slc16a2 cooperates with organic anion transporter Oatp1c1 (Slco1c1)^7^ or L-type amino acid transporter Lat2 (Slc7a8)^8^ in the mouse brain, raising the possibility that combinations of transporters extend control over additional developmental functions.

Auditory development is highly sensitive to thyroid hormone^9^. Hearing loss is associated with endemic iodine deficiency^10^, resistance to thyroid hormone^11^ and congenital hypothyroidism^12,13^. In rodents, the cochlear sensory epithelium, which contains the mechanosensory hair cells, is a major site of T3 action^14–16^. The *Thrb* thyroid hormone receptor gene promotes developmental remodeling of the sensory epithelium prior to the onset of hearing^17–19^. Thyroid hormone also influences hair cell survival, and cochlear functions including the endocochlear potential^19,20^ as well as the maintenance of hearing^21^.

The compartmentalized anatomy of the cochlea led us to hypothesize that membrane transporters are necessary to convey blood-borne hormone to its target tissues^22^. Blood enters the cochlea through the spiral modiolar artery then branches through radiating arterioles to capillary networks in the lateral wall before draining through collecting venules and the spiral modiolar vein^23^. These vessels bypass the sensory epithelium, implying a need for mechanisms that transport T3 and T4 internally. A need for transport is further implied by the requirement in auditory development for type 2 deiodinase, which amplifies levels of T3 by conversion from T4^22^. Type 2 deiodinase is expressed in the medial cochlea and lateral wall in proximity to blood vessels but distant from the sensory epithelium^24^. We previously reported expression of Slc16a2 and a related thyroid hormone transporter Slc16a10 (Mct10)^25^ in cochlear tissues^26^. Here we report deafness and loss of hair cells in mice lacking Slc16a2 and Slc16a10. Treatment with T3 partly rescued phenotypes, supporting a critical role for transmembrane transport of T3 for the development and maintenance of the cochlea.

## Results

### Auditory deficits in mice lacking Slc16a2 and Slc16a10

**Figure 1:**
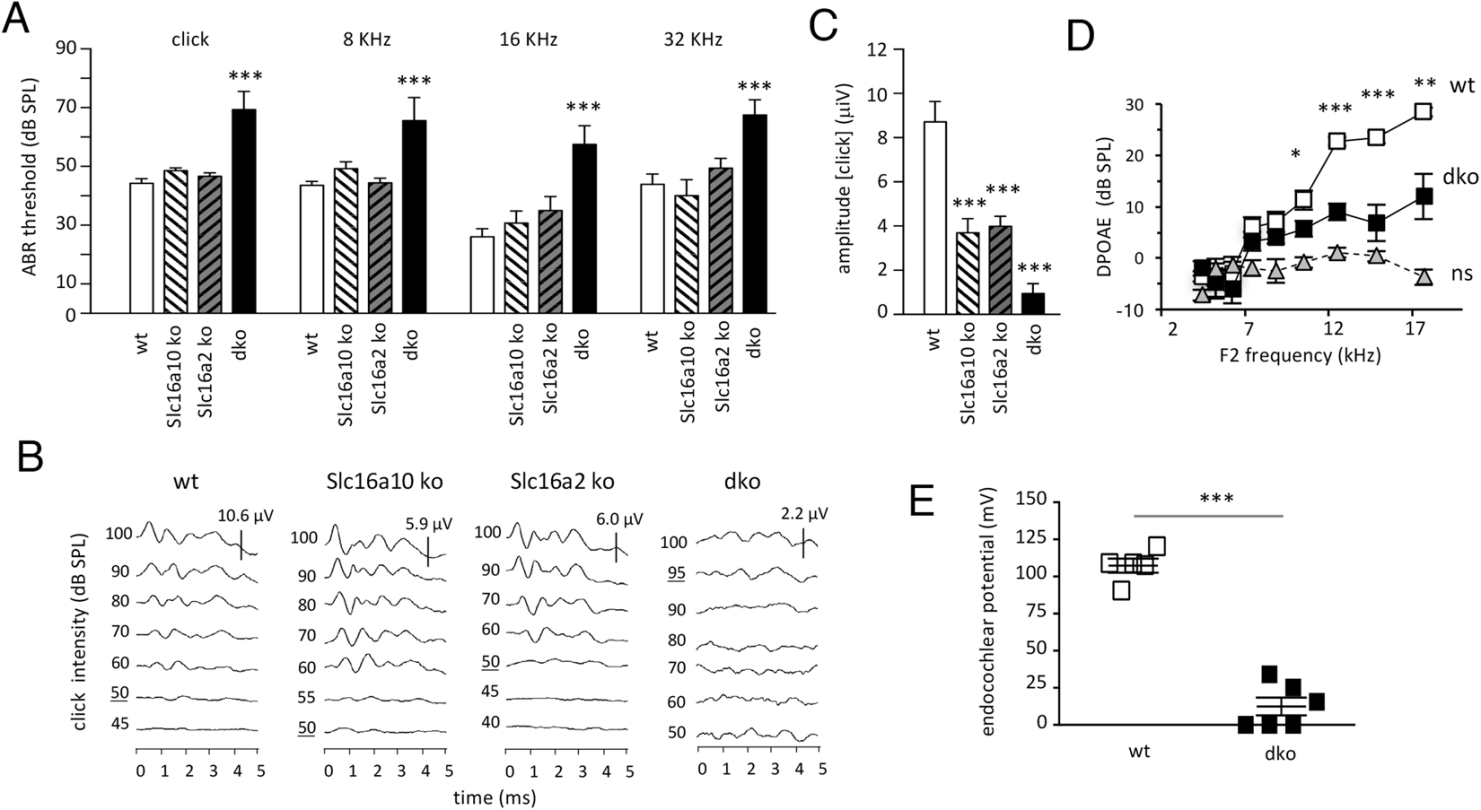
Auditory defects in mice lacking Slc16a2 and Slc16a10. (**A**) Mean thresholds for the auditory-evoked brainstem response (ABR) for click and pure tone stimuli. Groups of single and double (dko) knockout mice (n = 7 to 14) at 6–12 weeks of age. Comparison of mutants to wt used a one-way ANOVA followed by Bonferroni’s t-test; ***p < 0.0001 dko versus wt at each frequency. **(B)** Representative ABR waveforms for a click stimulus at different intensities showing lack of peaks in the dko. Thresholds underlined. Note different response scales (μV) for each genotype. (**C**) Mean amplitudes of responses to a click applied at equivalent high intensity (90 dB SPL) to each genotype. Amplitudes represent the first peak to following trough of waveforms. Groups of 3 wt and 4 to 6 mutants. Comparison of mutants to wt used a one-way ANOVA followed by Bonferroni’s t-test; ***p < 0.0001 for each genotype. **(D)** Distortion product otoacoustic emission (DPOAE) for adult mice. Groups of 7 mice. The dko was impaired compared to wt (Student’s t-test) for F2 frequencies above 10.5 kHz; ns, noise background. *p = 0.0299; **p = 0.0022; ***p < 0.001. (**E**) Endocochlear potential in 5 wt and 6 dko mice at 3 months of age. Three dko mice gave no detectable response. Student’s t-test, ***p < 0.001.

To investigate the requirement for Slc16a2 and Slc16a10 for hearing, we analyzed the auditory-evoked brainstem response (ABR). In Slc16a2- or Slc16a10-deficient mice, auditory thresholds were similar to those in wt mice at 6–12 weeks of age (approximately young adult ages). However, in double knockout (dko) mice lacking both transporters, thresholds were markedly elevated compared to wt mice (Fig. 1A), thus unmasking a critical combined role for these transporters for auditory function. Responses in dko mice were defective for a click (broad band of frequencies) or pure tone stimuli at frequencies of 8, 16 and 32 kHz that span the sensitive range of hearing in mice. Defects persisted in older dko mice (6–9 months old), indicating a permanent loss of hearing. Severe impairment was also evident in weanling dko mice (3–4 weeks old), indicating an early-onset of hearing loss (not shown).

Analysis of ABR waveforms showed that compared to wt mice, the residual responses that could be detected in dko mice were much diminished in magnitude (Fig. 1B). When subjected to a click stimulus at equivalent high intensity (90 dB sound pressure level), the mean amplitude of the first peak was severely reduced by ~90% in dko compared to wt mice (Fig. 1C). In ~50% of dko mice, no specific waveform could be detected for a click stimulus. Amplitudes were also reduced in Slc16a2 and Slc16a10 single mutants, suggesting that although thresholds were in the normal range, the magnitude of the response was subtly compromised in the absence of either transporter alone (Fig. 1C).

In addition to defects in the ABR, an overall measure of auditory function, dko mice also displayed impairment of the distortion product otoacoustic emission (DPOAE) (Fig. 1D), a measure of the function of cochlear outer hair cells during the response to sound. Furthermore, the endocochlear potential (EP), the positive potential difference in the endolymph in the scala media that is considered necessary for auditory transduction, was substantially reduced in adult dko mice compared to wt mice (Fig. 1E). These results indicate that cochlear defects contribute to hearing loss in dko mice.

### T4 and T3 levels in dko mice

**Figure 2:**
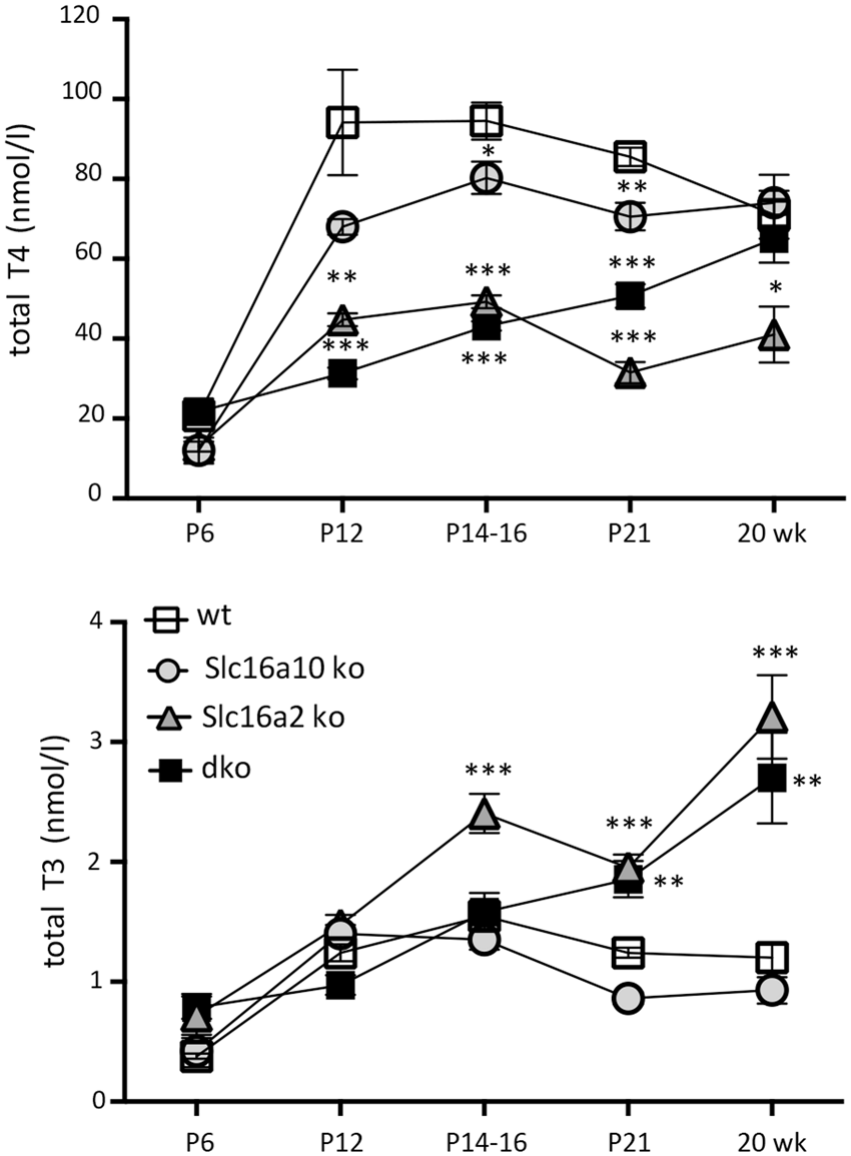
Serum T4 and T3 levels. T4 and T3 were measured at postnatal and adult (20 week) ages (mean ± sem). Comparison across all genotypes was determined using two-way ANOVA and Tukey’s post hoc analysis. For mutants versus wt, for T4 at P12: ***p < 0.001, **p = 0.0011; at P14–16: *p = 0.0277, ***P < 0.001; at P21: ***p < 0.001, **p = 0.0059; at 20 weeks: *p = 0.0416. For T3 at P14-16: ***p < 0.001; at P21: **p = 0.0013, ***p < 0.001; at 20 weeks: ***p < 0.001, **p = 0.0051.

We investigated levels of circulating thyroid hormone in dko mice as it is known that overt deficiency of thyroid hormone at early postnatal stages causes hearing loss in mice^27^. At P6, during the period of cochlear remodeling before hearing normally begins, serum levels of T4 and T3 were not significantly different in dko compared to wt groups (Fig. 2). In wt mice, T4 and T3 levels rise to peak between P12 and P16, then decline slightly after weaning^24,28^. In dko mice during this period, T4 was reduced, although only to a similar extent as in Slc16a2 mutant mice, which have minimal auditory defects. In adult 20-week old dko mice, T4 was normal and T3 was ~2-fold elevated. Similar trends for T4 and T3 have been reported for dko mice at 3 weeks of age, shortly after hearing normally begins^29^.

*Slc16a10* mutants displayed minimal changes in T4 and T3 compared to wt mice at any age examined. *Slc16a2* mutants, as reported previously^29,30^, displayed an approximately 50% decrease in T4 and an ~2-fold increase in T3 as adults and a similar trend at juvenile ages (P12 - P21). In summary, dko mice displayed levels of T4 and T3 that would normally be expected to suffice for the development of hearing. Moreover, changes in T4 and T3 in the dko, which have severe deafness, were more modest than in *Slc16a2* single mutants, which have minimal auditory defects. Thus, systemic hormonal changes are unlikely to explain the deafness in dko mice.

### Developmental and degenerative defects in the sensory epithelium

**Figure 3:**
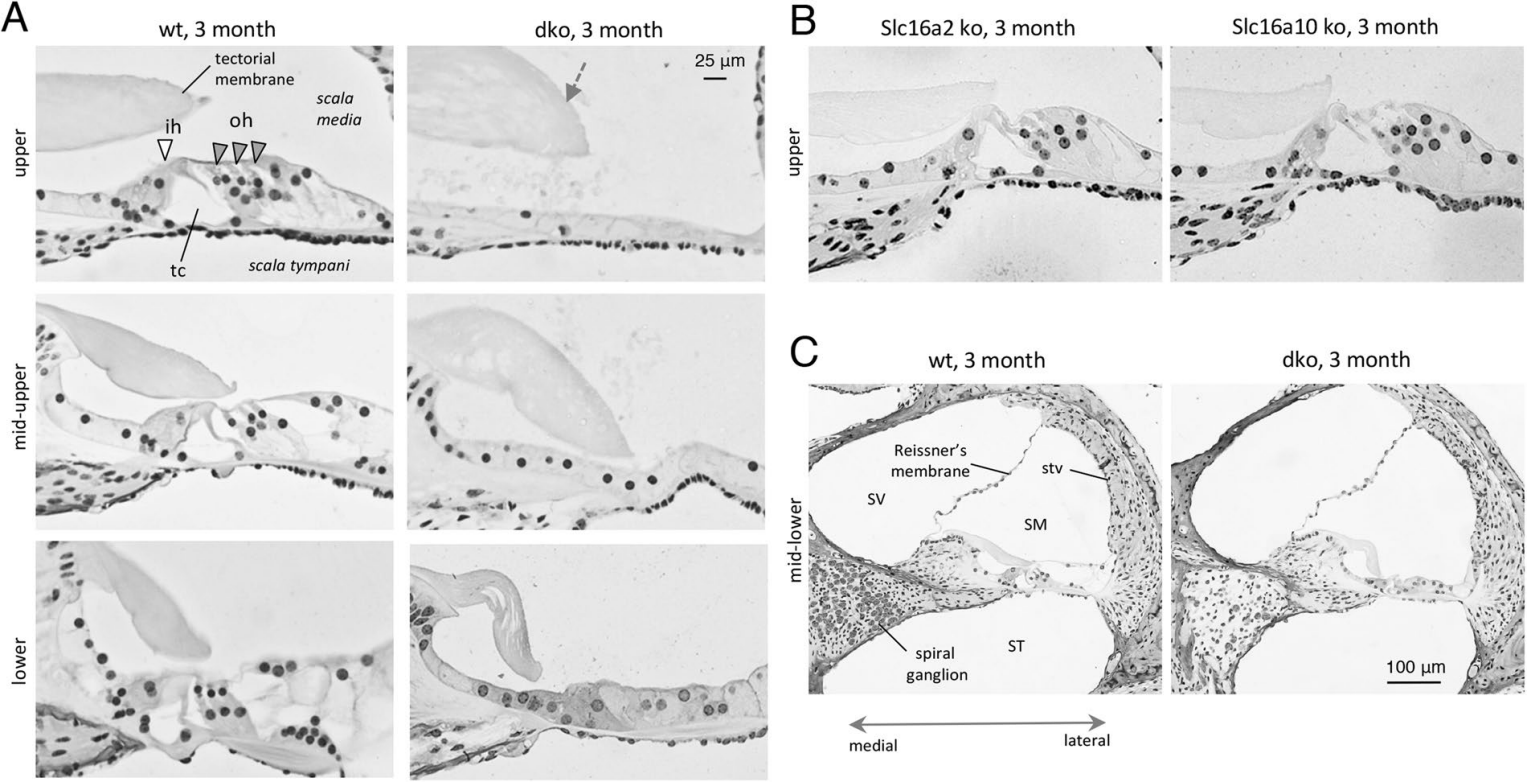
Degeneration of sensory epithelium in adult dko mice. (**A**) Histological sections showing degeneration of the sensory epithelium in dko mice at 3 months of age. Degeneration is most severe in upper cochlear regions with widespread loss of hair cells and support cells and a flattened epithelium. The dko has a somewhat swollen tectorial membrane (grey arrow). White and grey triangles mark inner hair (ih) and outer hair (oh) cells, respectively; tc, tunnel of Corti. (**B**) *Slc16a2* or *Slc16a10* single gene mutants displayed no overt abnormality. Representative upper regions of the cochlea are shown for comparison to the dko. Scale, same as in A. (**C**) Overview showing that the cochlear chambers (scala media, SM, scala tympani, ST, scala vestibuli, SV) and Reissner’s membrane appear histologically normal. The spiral ganglion shows loss of cells in the dko (more details in Fig. 5); stv, stria vascularis.

Histological examination of adult dko mice at 3 months of age revealed degeneration of the sensory epithelium with the most severe degeneration in upper (more apical) regions of the cochlear spiral (Fig. 3A). The dko mice displayed loss of outer hair cells, inner hair cells and support cells with a flattened residual epithelium. A more variable disorganization of the sensory epithelium was observed in lower (more basal) regions of the cochlea. The tectorial membrane, which extends over the inner sulcus to the hair cells and is critical for auditory transduction^31^, appeared moderately swollen, resembling a characteristic feature of hypothyroid mice^15,32^. *Slc16a2* or *Slc16a10* single mutants displayed no overt defects in cochlear morphology (Fig. 3B), suggesting that either transporter alone can promote and maintain cochlear structure, consistent with the minimal auditory impairment observed in either single mutant. In the dko, the overall structure of the cochlear chambers (scala media, scala tympani, scala vestibuli) was intact (Fig. 3C).

**Figure 4:**
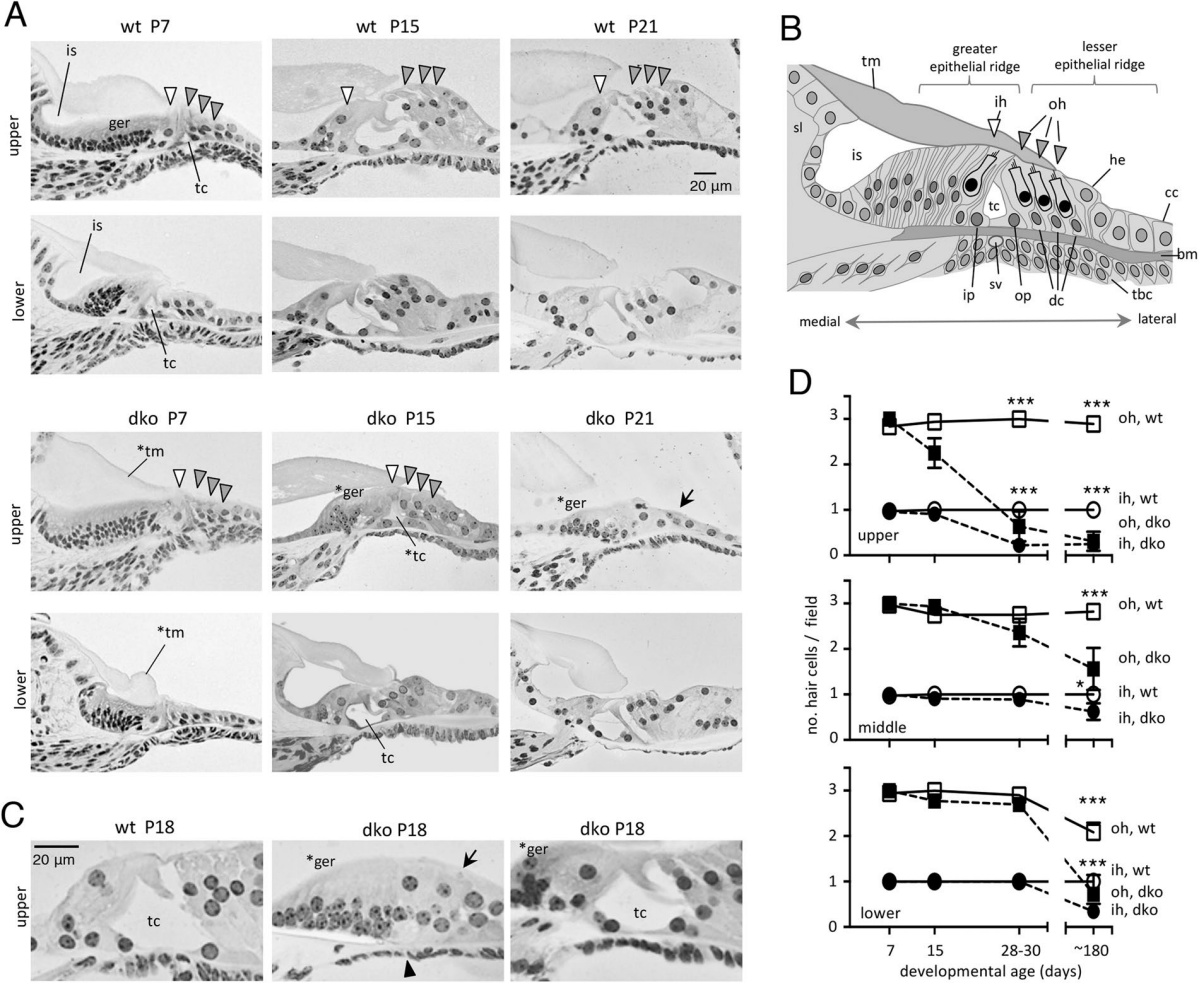
Developmental and degenerative cochlear abnormalities in dko mice. (**A**) At P7, the cochlea is being remodeled: in wt, the greater epithelial ridge (ger) is partly regressed and the inner sulcus (is) and tunnel of Corti (tc) are partly open. Lower regions are more advanced than upper regions because of the basal-apical progression of development. At P15, remodeling nears completion with upright, elongated hair cells (arrowheads) and open tc. The dko at P7 has hair cells and support cells but has almost no opening of the inner sulcus or tc and a malformed tectorial membrane (*tm). The dko is still retarded at P15 with limited regression of the ger (*ger) and poor opening of the tc (*tc), especially in upper regions. At P21, degeneration of the sensory epithelium (arrow) is also evident in upper regions. (**B**) Diagram of cell types of the immature cochlea (at ~P7). Abbreviations: bm, basilar membrane; cc, Claudius cells; dc, Deiters cells; he, Hensen cells; ih, inner hair cell; ip, inner pillar cell; is, inner sulcus; oh, outer hair cell; op, outer pillar cell; sl, spiral limbus; sv, spiral vessel; tbc, tympanic border cell; tc, tunnel of Corti; tm, tectorial membrane. The spiral vessel below the bm is prominent at birth and regresses during postnatal development^59^. (**C**) Higher magnification showing phenotypes in dko mice at P18. A mild example (right) shows delay in regression of ger cells (*ger) but an open tc, resembling wt (left). A severe example (middle) has delayed regression of the ger, a degenerated sensory epithelium (arrow) and absent tc (arrowhead). (**D**) Counts of outer (oh) and inner (ih) hair cells indicate the progression of degeneration in dko mice from P7 to P180 (6 months), determined on histological sections (mean ± sem). For wt versus dko at indicated ages: *p = 0.0396, ***p < 0.001.

At postnatal stages (P7) (Fig. 4A), the immature sensory epithelium of dko mice contained a normal complement of cell types including hair cells, indicating that the loss of cells observed at older ages arose from degeneration rather than developmental failure to form cell types. Figure 4B shows a reference diagram of cell types in the immature cochlea.

However, dko pups displayed retardation of cochlear remodeling (Fig. 4A), a defect typical of hypothyroid^15,20,32^ or *Thrb*-deficient^21^ mice. At P7, the sensory epithelium in wt mice begins to acquire mature morphology: the cell mass of the greater epithelial ridge regresses, the inner sulcus opens beneath the tectorial membrane and the tunnel of Corti opens between the pillar cells. These events progress in a basal-to-apical direction and thus are more advanced in lower than upper regions. In dko pups at P7, the greater epithelial ridge had not regressed, the inner sulcus and tunnel of Corti had not opened and the tectorial membrane was malformed. Remodeling was retarded in both lower and upper cochlear regions in dko mice at P7.

Remodeling did eventually progress in dko mice but lagged persistently behind the developmental status in wt mice. At P15 in wt mice, soon after the onset of hearing, the greater epithelial ridge had largely regressed, the hair cells and supporting cells had an upright structure and the tunnel of Corti was open. However, in dko mice, the greater epithelial ridge had only partly regressed, hair cells and supporting cells were poorly elongated and the tunnel of Corti was only partly open. The retardation was less pronounced in lower regions of the cochlea consistent with development occurring eventually in a basal-to-apical direction as in wt mice.

By P21, degeneration of the sensory epithelium was evident in upper cochlear regions in dko mice (Fig. 4A). In severe examples, the tunnel of Corti had collapsed and only sparse cells remained in the epithelium. The greater epithelial ridge retained an excessive cell mass, indicating persistently retarded development. In lower cochlear regions, degeneration was initially less obvious and remodeling had progressed to some extent to give a more normal morphology. Variable degeneration was also evident in dko mice at P18 (Fig. 4C). In summary, juvenile dko mice displayed a complex phenotype involving both retardation and a variable degeneration evident during the third postnatal week.

### Progression of degeneration

As an indicator of the long-term progression of degeneration of the sensory epithelium, hair cells were counted on histological sections up to 6 months of age (Fig. 4D). At P7 or P15, numbers of hair cells in the dko mice were not significantly abnormal in upper or lower cochlear regions. However, by P28-P30, in upper regions, numbers of outer and inner hair cells in dko mice were reduced by ~75% and at 6 months, by ~90% compared to wt mice. Loss of hair cells in middle and lower cochlear regions was less severe up to P28-P30 but was notable by 6 months at which stage ≥45% of outer and inner hair cells were missing.

### Morphology of the stria vascularis and spiral ganglion

**Figure 5:**
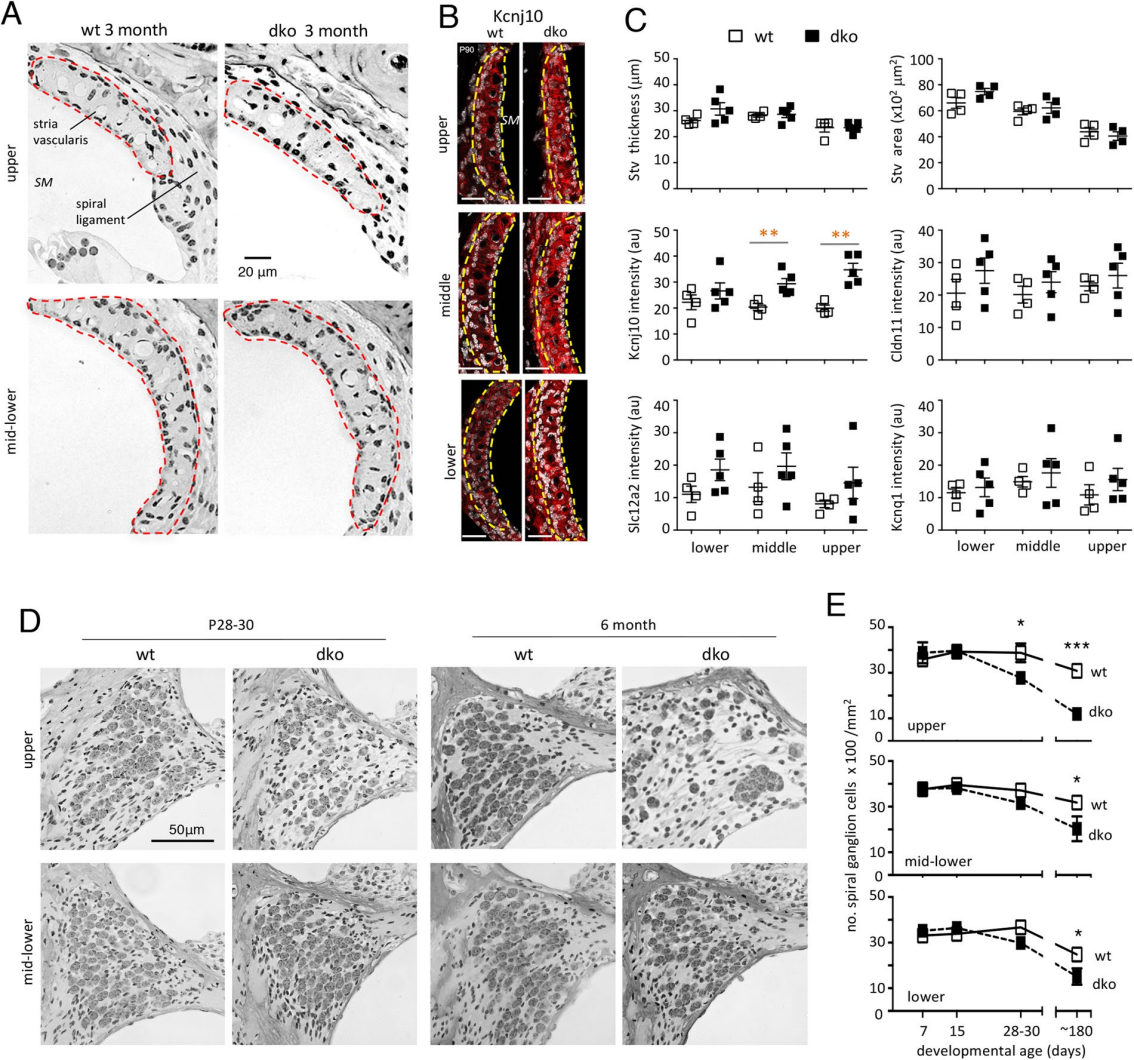
Morphology of the stria vascularis and spiral ganglion. (**A**) Histological sections showing the stria vascularis (red outline) and spiral ligament in adult wt and dko mice. SM, scala media. (**B**) Immunofluorescent analysis of the stria vascularis (yellow outline) showing enhanced signals for intermediate cell marker Kcnj10 in adult dko mice. DAPI staining of tissue (in grayscale). Scale bar, 20 μm. (**C**) Histomorphometry showing no change in area or thickness of the stria vascularis (Stv) in the dko. Immunofluorescence shows increased signals for Kcnj10 intermediate cell marker but not for marginal (Kcnq1, Slc12a2) or basal (Cldn11) cell markers. For wt versus dko, **p = 0.0096 for middle, **p = 0.0018 for upper region. (**D**) Sections showing loss of spiral ganglion cells in dko mice at juvenile and adult ages. Loss is most evident in upper regions. (**E)** Counts showing progressive loss of spiral ganglion cells from P7 to P180 (6 months). For wt versus dko at indicated ages: *p = 0.0463; ***p = 0.001 in upper region; *p = 0.0205 mid-lower region; *p = 0.0114 lower region.

Given the defective endocochlear potential, we investigated the stria vascularis, which has a role in maintaining homeostasis in the endolymph in the scala media^33^. No major histological abnormality was evident in the stria vascularis or adjacent fibrocyte layer of the spiral ligament (Fig. 5A). The area and thickness of the stria vascularis measured on cochlear sections were in the normal range (Fig. 5C). Analysis for markers of marginal (Kcnq1, Slc12a2), intermediate (Kcnj10) and basal (Cldn11) cell types of the stria vascularis indicated the presence of all three cell layers. However, the intensity of the Kcnj10 signal was increased in the dko mice compared to wt in middle and upper regions of the cochlea (Fig. 5B,C) suggesting an abnormality in the status of the stria vascularis. Reissner’s membrane was not obviously distorted, suggesting that fluid pressure in the scala media or scala vestibuli was not grossly abnormal (Fig. 3C).

The dko mice also began to lose spiral ganglion cells concomitantly with loss of hair cells after ~2 weeks of age (Fig. 5D,E). By P28, in upper regions of the cochlea in dko mice, almost 30% of spiral ganglion cells were lost and by 6 months, ~60% were lost compared to wt mice. By 6 months, a 36% loss of spiral ganglion cells was also evident in mid-lower regions of the cochlea.

### Rescue of cochlear remodeling by exogenous T3

To test the hypothesis that the cochlear phenotypes in dko mice result from insufficient T3 reaching target tissues, the T3-dependence of phenotypes was determined by administration of exogenous T3. The experiments were based on the assumption that high doses of T3 would overcome impaired uptake by tissues through utilization of other routes, potentially involving lower affinity types of thyroid hormone transporters.

**Figure 6:**
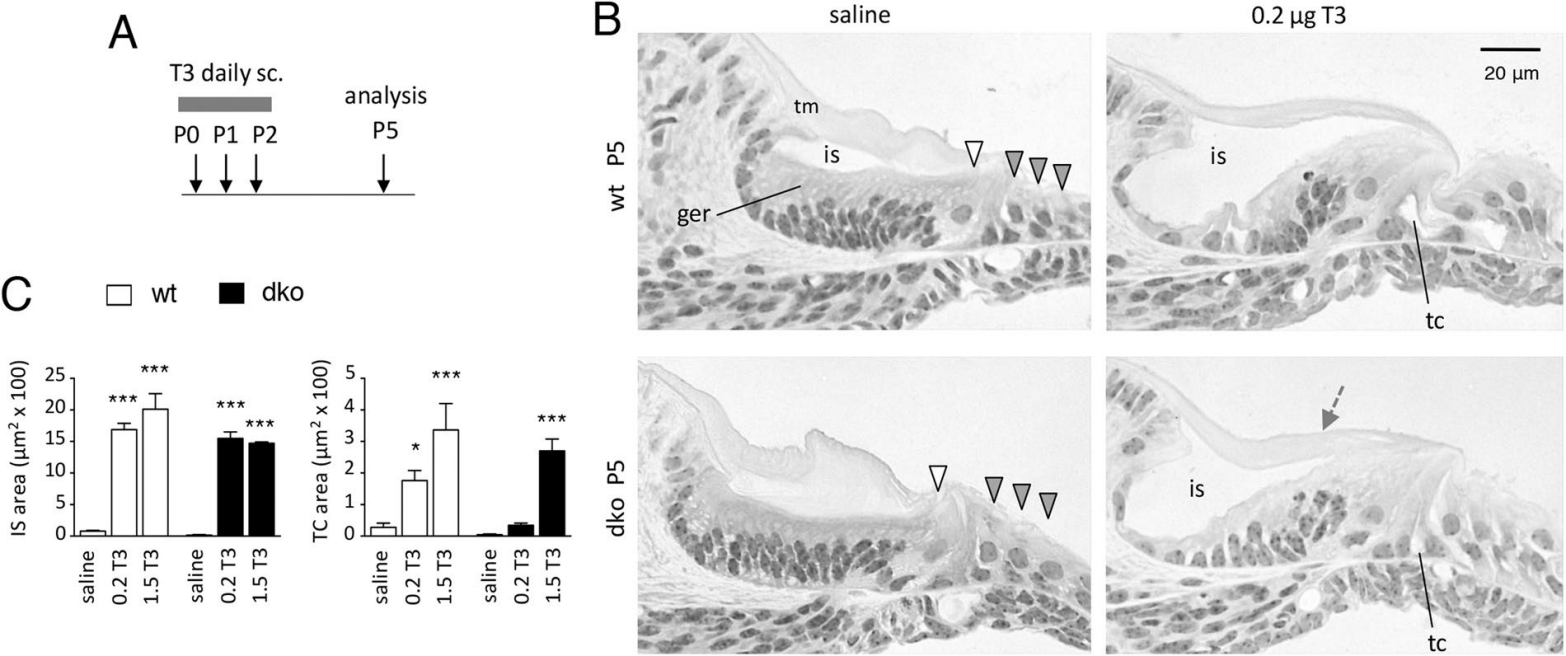
Rescue of cochlear remodeling by T3 treatment. (**A**) Scheme of administration of T3 (or saline vehicle) by sc injections at P0-P2 then analysis at P5. (**B**) Histological sections of the mid-lower cochlea showing status of remodeling. T3 but not saline stimulates opening of the inner sulcus (is) and tunnel of Corti (tc), regression of the greater epithelial ridge (ger) and a thinner tectorial membrane (tm, grey arrow) in both dko and wt mice. (**C)** Measurements of open areas of the inner sulcus (IS) and tunnel of Corti (TC) after treatment with saline or T3 given at 0.2 or 1.5 μg/day. Measured on middle regions of the cochlea on plastic sections using ImageJ. Groups were compared to saline-treated wt mice following a two-way ANOVA; *p = 0.0105; ***p < 0.001.

To investigate the developmental retardation of the cochlea, dko pups were injected with T3 or saline vehicle for three consecutive days at P0-P2, then examined histologically at P5 (Fig. 6A). In wt pups, as expected^34^, T3 accelerated the regression of the greater epithelial ridge and the opening of the inner sulcus and tunnel of Corti (Fig. 6B). In dko pups, T3 similarly stimulated regression of the greater epithelial ridge, partial opening of the inner sulcus and tunnel of Corti and thinning of the tectorial membrane. T3 recovered a similar degree of opening of the inner sulcus in dko pups as in wt pups (Fig. 6C). T3 at higher doses also stimulated opening of the tunnel of Corti in dko pups to a similar extent as in wt pups.

### Preservation of the sensory epithelium by exogenous T3

**Figure 7:**
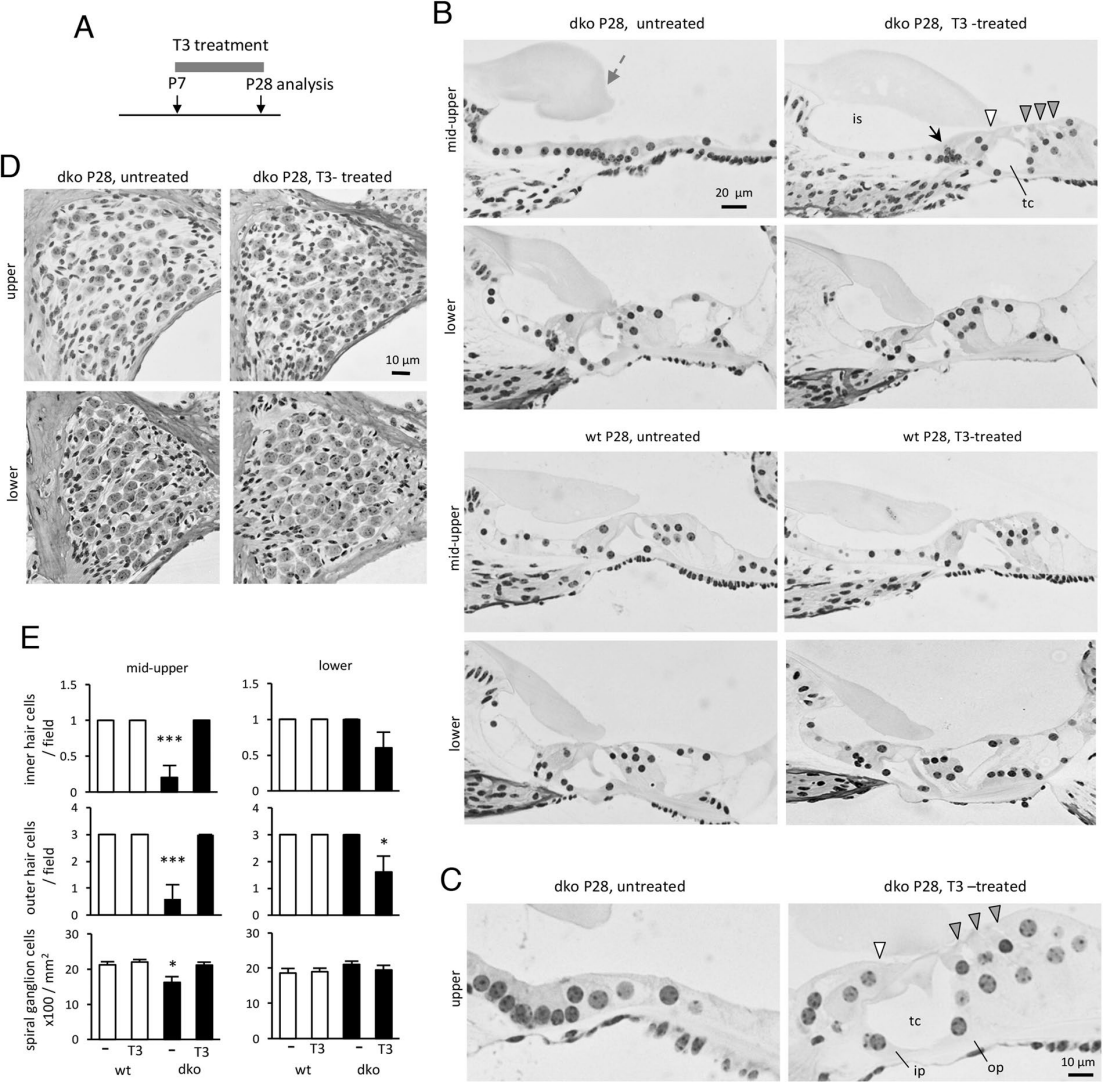
Preservation of cochlear morphology by T3 treatment. **(A**) Scheme of treatment from P7 to P28 then analysis at P28. T3 was given in drinking water. (**B**) Sections of mid-upper and lower regions of the cochlea after treatment. In the dko, the extensive degeneration in mid-upper regions is prevented by T3; the tectorial membrane is less swollen (grey arrow). A cluster of cells near the inner hair cell indicates persistent delay in regression of the ger (black arrow). In lower regions, there is little overt degeneration at this age. In wt mice, the sensory epithelium appears normal regardless of T3 treatment. (**C**) High magnification of upper cochlear regions in dko mice showing that T3 restores inner (ip) and outer pillar (op) cells, support cells and hair cells (inner and outer, white and grey arrowheads, respectively). The tunnel of Corti (tc) is open. **(D)** Sections showing partial recovery of spiral ganglion cells in the dko after T3 treatment. **(E)** Counts of hair cells and spiral ganglion cells at P28 following T3 or no treatment, determined on sections. Groups were compared to untreated wt mice following a two-way ANOVA: outer hair cells lower, *p = 0.0178; spiral ganglion cells mid-upper, *p = 0.0363; inner and outer hair cells mid-upper, ***p < 0.001.

The degeneration of the sensory epithelium in dko mice at more mature ages was preventable by administration of T3 over a later time course (Fig. 7A). T3 was given by continuous provision in the drinking water from P7 until analysis at P28. Previous studies demonstrated that exogenous T3 administered in the first few postnatal days produces long term deafness whereas T3 given at P7 or later does not cause hearing loss^34^. Thus, treatment starting at P7 was not expected to cause additional auditory damage.

In wt mice with or without T3 treatment, no loss of hair cells nor malformation of the sensory epithelium was observed at P28 (Fig. 7B). In untreated dko mice, as expected, the epithelium was flattened and lacked hair cells, especially in upper cochlear regions. However, in dko mice, T3 treatment remarkably recovered a more normal cochlear morphology. Inner and outer hair cells, pillar cells and other supporting cell types were restored and the sensory epithelium regained an upright structure with an open tunnel of Corti (Fig. 7B,C). A small cluster of cells remained near the base of the inner hair cells suggesting that T3 treatment started at P7 was unable to correct fully the early regression of the greater epithelial ridge in dko mice. Counts on histological sections showed that T3 prevented loss of inner and outer hair cells (Fig. 7E). These results were confirmed in an independent experiment using an alternative route of administration of T3 by daily injection over the same time-course (not shown, see Methods). T3 given from P7 to P28 also recovered spiral ganglion cell numbers in the dko mice (Fig. 7D,E).

### Auditory function after treatment with exogenous T3

**Figure 8:**
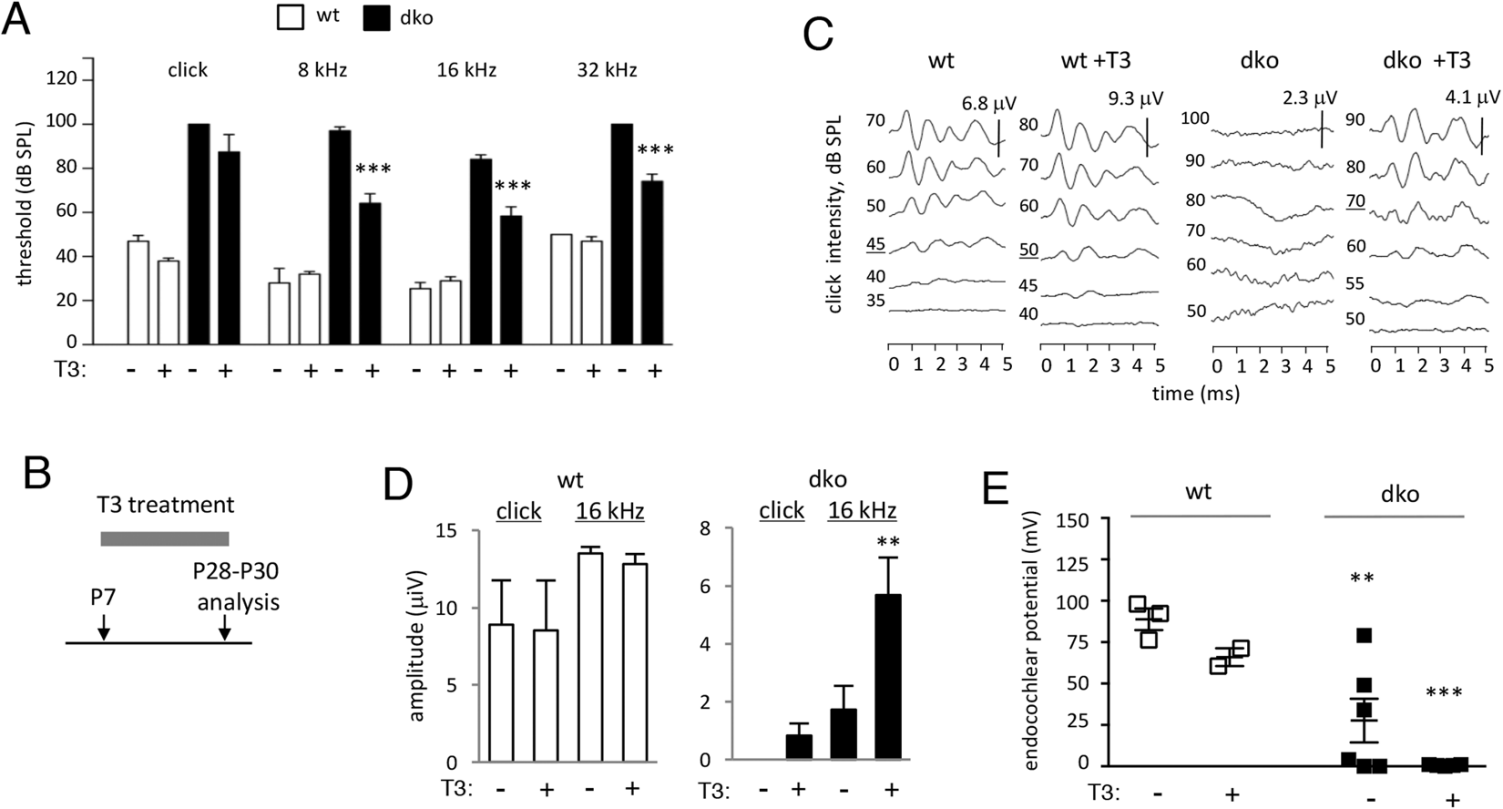
Auditory responses in dko mice after T3 treatment. (**A**) ABR mean thresholds at P28 after treatment with T3 in drinking water from P7 to P28. Groups contained 5 wt and 6 dko. Comparisons were made within genotype by two-way ANOVA; ***p < 0.001. (**B**) Scheme for treatment with T3. (**C**) Enhanced definition and amplitudes of ABR peaks in T3-treated mice; representative traces for a click stimulus. Note different scale bars (μV) for wt and dko. (**D**) Waveform amplitudes (mean ± sem) in wt and dko mice. Amplitudes were determined for peak 1 to the following trough for a click and representative pure tone (16 kHz) stimulus. T3 made little difference in wt mice (p > 0.3) but enhanced amplitudes in dko mice for 16 kHz (**p = 0.0022) and a click (but not statistically significant, p = 0.5602). Groups of 3 untreated and 5 treated wt; 5 untreated and 6 treated dko. **(E**) Endocochlear potential in dko mice was not restored by treatment with T3. The endocochlear potential was significantly reduced in untreated dko (**p = 0.01) and T3 treated dko (***p < 0.001) compared to wt untreated mice at this age, similar to the defect in adult mice.

To determine if T3 treatment also recovered auditory function in dko mice, the ABR was analyzed at P28 following administration of T3 in drinking water from P7 to P28 (Fig. 8A,B).

In wt mice, as expected for treatment over this period^34^, T3 produced little or no change in ABR thresholds compared to untreated mice (Fig. 8A). In dko mice, T3 stimulated a limited recovery of the ABR. Thresholds were slightly lower in T3-treated than untreated dko groups across a range of frequencies. Figure 8C shows representative waveforms for a click stimulus in which the untreated dko lacked discernible peaks, even with high intensity stimuli (up to 100 dB SPL) whereas the T3-treated dko displayed distinct peaks. Similar improvements in the definition and amplitude of the waveform in dko mice were observed for pure tone stimuli. The mean amplitude of response was unchanged by T3 in wt groups but was enhanced in dko groups, as shown in Fig. 8D for a click and a representative pure tone stimulus (16 kHz) when delivered at equivalent high intensity (90 dB SPL).

Mice treated with T3 in the same way (in drinking water from P7) were also tested for recovery of the endocochlear potential at P28 - P30 (Fig. 8E). Compared to wt mice, untreated dko mice at ~P30 displayed a reduced endocochlear potential resembling the defect in adult dko mice (see Fig. 1E). T3 did not rescue the endocochlear potential in dko mice at P28 - P30. Thus, the endocochlear potential in dko mice was not restored by treatment with T3 under conditions that rescued hair cell survival and partly rescued the ABR.

## Discussion

This study demonstrates a key role for Slc16a2 (Mct8) and Slc16a10 (Mct10) transporters in the development of hearing and maintenance of cochlear structure. The findings suggest that apart from the systemic provision of thyroid hormone, cellular uptake or efflux of the hormone by transmembrane transporters is required to promote cochlear development and hearing.

Studies of Allan-Herndon-Dudley syndrome and of mice with Slc16a2 mutations have established a requirement for Slc16a2 for T3 action in the brain and pituitary-thyroid axis but less is known about Slc16a10. In mice, Slc16a10 facilitates transport of aromatic amino acids in liver and kidney^35^, consistent with initial descriptions of this protein as a T-type amino acid transporter^36^. However, Slc16a2 and Slc16a10 share a related transmembrane structure and ~53% amino acid identity, which led to the finding that Slc16a10 also transports T3 and less efficiently T4, across the plasma membrane of cells *in vitro*^25,37,38^. Deletion of Slc16a10 in Slc16a2-deficient mice modifies the levels of T4 and T3 in serum, kidney and brain, suggesting that both transporters cooperate in a complex manner to determine the thyroid hormone status of certain tissues^29^.

Our study reveals a new role for Slc16a10 in the auditory system and suggests that this role, at least in part, involves transport of T3. First, the retarded cochlear morphology in dko mice resembles that of hypothyroid^15^ or *Thrb*-deficient^21^ mice. Secondly, cochlear morphology in the dko is substantially rescued by administration of T3. Thus, although Slc16a10 can transport aromatic amino acids and T3 *in vitro*, the particular substrate utilized *in vivo* may depend on tissue and developmental context. Conceivably, the substrate specificity is governed in part by the co-expression of other types of transporters, such as Slc16a2 in cochlear cell types.

Overt hearing loss arises only in the absence of both transporters indicating that Slc16a10 and Slc16a2 compensate functionally for each other in the auditory system. The simplest explanation may be direct substitution since *in situ* hybridization^26^ revealed partly overlapping expression of both transporters in the inner sulcus, sensory epithelia, Claudius cells and tympanic border cells. The developmental expression profiles are complex with *Slc16a10* following *Slc16a2*. The induction of *Slc16a10* at ~P5 may provide a temporal cue for the remodeling of the cochlea. Type 2 deiodinase, a T3-amplifying enzyme that is required for cochlear development^22^, is also induced in the cochlea at this time^24^ suggesting that coordinated mechanisms drive the T3-dependent remodeling that precedes the onset of hearing. However, the level of Slc16a10 is low and detailed analysis at cellular resolution awaits improved methods to detect the protein.

The solute carrier Slc16A family, includes 14 members and was originally named a monocarboxylate transporter (Mct) family as several members transport monocarboxylate substrates such as lactate and pyruvate^39^. Among this family, Slc16a10 and Slc16a2 constitute a distinct subgroup based on protein sequence and substrate preference for iodothyronines (T4 and T3)^25^. Our finding of cooperation for these transporters in the auditory system together with previous evidence in the endocrine system^29^ raise the possibility that Slc16a2 and Slc16a10 together may be found to mediate additional functions for thyroid hormone *in vivo*.

In the cochlea, we proposed that different transporters demarcate a cellular route that transfers thyroid hormone from the bloodstream to tissues within the cochlea^26^. The phenotype of the dko mice supports a role for Slc16a2 and Slc16a10 in transport of T3 in sensory or adjacent tissues, in which expression of the transporters overlaps partly with that of the *Thrb* receptor gene^21^. The initial steps for uptake of T4 and T3 from the circulation may involve other types of transporters including the L-type amino acid transporter Lat1 in endothelial cells and the organic anion transporting polypeptide Oatp1c1 (Slco1c1) in tissues adjacent to capillaries in the spiral ligament and modiolus^26^.

The expression of Slc16a2 and Slc16a10 in Claudius cells and other support cells leads to speculation that these cells might form a conduit for relaying hormone internally from the lateral wall or medial cochlea to the sensory epithelium. Slc16a2 and Slc16a10 mediate both uptake and efflux of T3^25^, which may allow cell-to-cell relay of hormone. Interestingly, other intercellular transport functions have been attributed to Claudius, Deiters, Hensen and root cells and another more medial cellular network in the spiral limbus and inner sulcus. These cells form epithelial cell gap junctional networks^40^ that may transfer potassium ions away from the hair cells to the lateral wall of the cochlea^41^. The transport of ions, metabolites and nutrients by specialized epithelial cells maintains homeostasis in the cellular and fluid compartments of the cochlea^33^. Our findings suggest that cell-mediated transport of hormones may be similarly critical to cochlear function.

Deafness in the dko is severe and is comparable to that caused by deletion of all T3 receptors^19^ suggesting that Slc16a2 and Slc16a10 together transport most of the thyroid hormone required by the immature cochlea under normal conditions. However, the rescue experiments imply that other lower affinity thyroid hormone transporters are utilized when T3 is given in excess. In common with proposals for the brain^2,42^, the cochlea may express different classes of transporters: high specificity types such as Slc16a2 and Slc16a10 and other types, perhaps with a wider spectrum of substrates that may not normally transport thyroid hormone.

Allan-Herndon-Dudley syndrome is associated with impaired speech but there have been few remarks on hearing, perhaps reflecting a lack of obvious defects or lack of detailed analysis in some cases. One report has mentioned hearing impairment^3^ but others have not^43^. It is conceivable that as in mice, compensation by SLC16A10 masks auditory defects in patients with SLC16A2 (MCT8) mutations. Nonetheless, subtle defects in auditory function or auditory processing might contribute to defective speech in this syndrome. Mutations in human SLC16A10 have not been reported.

Our study indicates a remarkable ability of T3 to rescue cochlear structure and hair cell survival in dko mice. Degenerative forms of hearing loss are common in human populations and may result from many environmental or genetic factors^44^. Regarding possible therapeutic approaches to deafness, much effort has focused on hair cells because these cells cannot regenerate naturally in mammals^45^. Our results suggest that T3 may be worth testing in attempts to preserve hair cells in experimental models of deafness. The pattern of hair cell loss in the dko mice with an initial bias to upper cochlear regions is unusual and has not been observed in hypothyroid or *Thrb*-deficient mice. On the contrary, *Thrb*-deficient mice display most hair cell loss in the lower cochlea^21^, suggesting that unknown factors contribute to the regional patterns of degeneration. In dko mice, hair cell loss might be a secondary defect as auditory function was impaired at all frequencies tested and did not obviously correlate with tonotopical domains along the length of the cochlea.

The loss of endocochlear potential would impair hearing in dko mice and may reflect defects in the tissues of the lateral wall^33^. Interestingly, dko mice display elevated expression of *Kcnj10* in the intermediate cells of the stria vascularis. In hypothyroid mice, the expression of *Kcnj10* is reduced in the stria vascularis^20^ and the cerebral cortex^46^, consistent with this gene being T3-responsive. The increased expression of *Kcnj10* in the stria vascularis in dko mice may result from the elevated T3 levels in the dko. *Kcnj10* is required for an endocochlear potential in mice^47^ and mutations in *KCNJ10* occur in a syndromic form of human deafness^48^. T3 treatment recovered cochlear morphology and a limited ABR but not endocochlear potential. The persistent defect in endocochlear potential may explain why T3 did not rescue auditory function to a greater extent. The results also support a view that the survival of the sensory epithelium is not strictly dependent on the endocochlear potential^49^.

It is possible that in dko mice, early events that promote the endocochlear potential are disrupted before the beginning of T3 treatment at P7, or that no alternative transporters for T3 are present in tissues that generate the endocochlear potential. A role for T3 in determining the endocochlear potential is consistent with a partial loss of endocochlear potential reported in mice lacking thyroid hormone receptors^19^ and in hypothyroid dwarf mice^20^. T3 may act directly in lateral wall tissues that contribute to the endocochlear potential as the *Thrb* gene is expressed in root cells and fibrocytes of the spiral ligament^21^, as well as in the sensory epithelium and spiral ganglion. It is not excluded that the loss of endocochlear potential reflects impaired transport of substrates other than T3.

Finally, we note that the complex auditory phenotype of dko mice may involve additional defects not in the sensory epithelium or lateral wall, as T3 also influences the spiral ganglion^26,50^ and brainstem auditory nuclei^51^.

## Methods

### Mouse Strains

The null allele of *Slc16a2* (*Mct8)* was previously reported^52^. The *Slc16a10* (*Mct10)* null allele carries a premature stop at codon 88 (Y88*), which truncates most of the protein including 11 of 12 transmembrane domains^35^. Double mutant (dko) mice were originally on a C57BL/6 (Charles River) background^29^ and at NIH were further backcrossed for up to 2 generation onto a C57BL6/J (Jackson Lab) background. *Slc16a2*-deficient groups were generated by crossing *Slc16a2*+/− females with y/− males. *Slc16a10*-deficient groups were generated by crossing *Slc16a10*+/− males and females. Groups of dko mice were generated by crossing *Slc16a10*+/−; *Slc16a2*+/− females with either *Slc16a10*+/−; *Slc16a2*y/− males or *Slc16a10*−/−; *S lc16a2*y/− males. Controls were wt littermates from these crosses. For some T3 treatment experiments, wt progeny were intercrossed to generate additional wt mice on the same genetic background. Genotypes were determined by PCR as described^35,52^. Most analyses were performed on mixed groups of males and females in approximately equal numbers. Male-only groups were used for hormone measurements. Animal studies were conducted under approved protocols at NIDDK and NIDCD at NIH and Minnesota State University Mankato. All methods were performed in accordance with relevant guidelines and regulations.

### Thyroid hormone treatment

T3 (Sigma-Aldrich) was provided to pups in the drinking water or by daily sub-cutaneous injection in the nape of the neck, as described^22,53^ for the time courses noted in Figs 6, 7 and 8. Previous studies reported that thyroid hormone is transferred through the milk of nursing dams at neonatal stages^54^. Short term postnatal treatment involved injections on P0, P1 and P2 with 0.2 μg or 1.5 μg T3 in saline or with saline alone in a 10 μl volume. Longer term treatment was given by injections from P7 to P28-P30 with 0.5 μg T3 in saline or saline alone in a 10 μl volume (4–11 mice), or by T3 added in drinking water at 0.5 μg/mL from P7 to P28 (5–6 mice).

### Auditory Function Tests

The auditory-evoked brain stem response (ABR) was analyzed using a SmartEP system (Intelligent Hearing Systems, Miami, FL) as described^53^. Mice were anesthetized with tribromoethanol (avertin) (0.25 mg/g body weight). Active, reference and ground electrode needles were placed subcutaneously at the vertex, ventrolateral to the left ear, and ventrolateral to the right ear, respectively. Groups (mixed males and females) at 6–12 weeks of age, contained 14 wt, 7 Slc16a10 ko, 8 Slc16a2 ko and 8 dko mice. Groups at 3–4 weeks of age contained 7 wt and 9 dko. Groups at 6–9 months of age contained 10 wt and 10 dko. For dko mice with no detectable response, a threshold was assigned at 100 dB SPL, the maximum stimulus level possible. The Distortion Product Otoacoustic Emission was analyzed using a Smart DPOAE system (Intelligent Hearing Systems) on 8 week old mice (groups of 7) under avertin anesthesia, as described^53^.

Methods for endocochlear potential measurement have been previously described by Wangemann and colleagues^55,56^. Briefly, groups (at ~P90 and ~P28-P30) were anesthetized with avertin (0.35 mg/g body weight). Endocochlear potential measurements were made using glass microelectrodes inserted into the round window and through the basilar membrane of the first turn of the cochlea. Induction of anoxia, allowing measurement in the anoxic-state, was accomplished by intramuscular injection of succinylcholine chloride (0.1 µg/g body weight) after establishment of deep anesthesia followed by additional injection of avertin. Measurement in the anoxic-state provides an indicator of the lowest endocochlear potential and hair cell function: in the presence of functional hair cells, the anoxic-state endocochlear potential is negative, whereas the endocochlear potential is zero if the hair cells are not functional. In all adult dko mice at ~P90, bony overgrowth of the bulla was noted and clear fluid was observed to arise from the region of the round window in a continuous fashion prior to insertion of the electrode for recording the endocochlear potential. Data were recorded digitally (Digidata 1440 A and AxoScope 10; Axon Instruments) and analyzed using Clampfit10.

### Measurement of Thyroid Hormones

Serum T4 and T3 levels were determined from trunk blood samples collected from male mice by radioimmunoassay (RIA) as described^57^. Hormone values represent individual mice. Groups contained 3–12 male mice per genotype at P12, P14–16, P21, and 20 weeks of age. At P6, groups of 3–5 mice per genotype were measured as pools consisting of 3 mice. Hormone values at P21 have been previously published and are included in Fig. 2 for completeness^29^.

### Histology

Cochleae were dissected from the temporal bone, fixed overnight in phosphate buffered saline (PBS) containing 3% glutaraldehyde/2% paraformaldehyde, decalcified in 0.1 M ethylenediaminetetraacetic acid (EDTA) in PBS, dehydrated through a graded series of ethanol concentrations (50, 70, 80, 95, 100%) then embedded in glycol methacrylate plastic (Polysciences, Warrington, PA) as described^53^. Four μm thick sections in the vertical, mid-modiolar plane were stained with hematoxylin Gill #3 (Sigma), air-dried, then coverslipped in Permount (Sigma-Aldrich). At least one cochlea/mouse for ≥3 mice was analyzed at each age.

### Immunofluorescence

For immunohistochemistry of the lateral wall, adult inner ears were fixed, then decalcified in 150 mM EDTA for 5–7 days, transferred to 30% sucrose, embedded then frozen in SCEM tissue embedding medium (Section-Lab Co, Ltd.; Hiroshima, Japan). Adhesive film (Section-Lab Co, Ltd.; Hiroshima, Japan) was fastened to the cut surface of the sample in order to support the section and cut slowly with a blade to obtain 6 µm thickness sections. The adhesive film with section attached was submerged in 100% ethanol for 60 seconds, then transferred to distilled water. The adhesive film consists of a thin plastic film and an adhesive and prevents specimen shrinkage and detachment. This methodology allows for high quality anatomic preservation of the specimen and allows sectioning at reduced thickness (to 0.5 µm). Mid-modiolar sections were obtained for samples in which an endocochlear potential recording had been performed.

For analysis of stria vascularis markers, mid-modiolar sections were washed in PBS then permeabilized and blocked for 1 hour at room temperature in PBS with 0.2% Triton X-100 (PBS-T) with 10% fetal bovine serum. Samples were incubated with primary antibodies in PBS-T with 10% fetal bovine serum, followed by three rinses in PBS-T, then incubated with AlexaFluor-conjugated secondary antibodies (1:250, Life Technologies) in PBS-T for 1 hour at room temperature. Where indicated, 4,6-diamidino-2-phenylindole (DAPI, 1:10,000, Life Technologies) was included with secondary antibodies to detect nuclei. Sections were rinsed in PBS three times and mounted in SlowFade (Invitrogen), then imaged using a Zeiss LSM 710 confocal microscope. Sections were mounted with SCMM mounting medium (Section-Lab Co, Ltd.; Hiroshima, Japan). Primary antibodies included rabbit anti-Kcnj10 (Alomone Labs, Cat# APC-035, polyclonal, dilution 1:200), rabbit anti-Cldn11 (Life Technologies, Cat# 364500, polyclonal, dilution 1:200), goat anti-Slc12a2 (Santa Cruz Biotech, Cat# sc-21545, polyclonal, dilution 1:200), goat anti-Kcnq1 (Santa Cruz Biotech, Cat# sc-10646, polyclonal, dilution 1:200), Phalloidin AlexaFluor 647 (Invitrogen, Cat# A22287, dilution 1:250).

### Cell Counts and Histological Measurements

Images were acquired for upper, middle and lower turns of the cochlea on 4 μm thick methacrylate sections in the mid-modiolar, vertical plane. The most extreme apical and basal regions were avoided. For developmental studies, hair cell and spiral ganglion cells were counted on 4 cochlear sections per mouse (groups of 4–11) at P7, P15, P28–30 and ~P180. Spiral ganglion cell counts were normalized relative to the surrounding area of Rosenthal’s Canal using the ImageJ program. For T3 treatment experiments, spiral ganglion cells were counted on 1–3 cochlear sections per mouse (groups 3–4 mice). Areas of the inner sulcus and tunnel of Corti were measured on middle turns of the cochlea on 3 sections per cochlea (groups of 3–7 mice) using ImageJ software.

ImageJ was utilized to calculate the cross-sectional area and thickness of the stria vascularis on mid-modiolar sections. Fluorescence intensity quantification was performed in ImageJ by calculating the fluorescence intensity of the outlined region of the stria vascularis on maximal intensity projection images. Fluorescence intensity was normalized by comparing the stria vascularis fluorescence intensity to that of a corresponding region in the scala media. Measurements were made for upper, middle, and lower turns of the cochlea for Kcnq1, Slc12a2, Kcnj10 and Cldn11 markers.

### Statistical Analyses

Unless stated otherwise, all values represent mean ± SEM. For pairwise comparisons (between wt and a given mutant), an unpaired 2-tailed Student’s t-test was used. For comparison of multiple genotype groups, ANOVAs were performed. For analysis of auditory thresholds, a one-way ANOVA followed by a Bonferroni’s posthoc t-test was used for each stimulus frequency. For comparison of hair cell and spiral ganglion cell numbers in wt and dko groups over developmental time or with T3 treatment, a two-way ANOVA followed by a Bonferroni’s posthoc t-test was used. For consistency with previous analyses of T4 and T3^58^, T4 and T3 levels were compared across genotypes using a two-way ANOVA and Tukey’s post hoc analysis. All statistical analyses were performed using GraphPad Prism version 6.0 h for Mac. The datasets generated and/or analyzed during the current study are available from the corresponding authors on reasonable request.
